# A Novel Puncture Method Combining Intracardiac Echocardiography and a 3D Mapping System for Catheter Ablation After a Lateral Tunnel Fontan Operation

**DOI:** 10.1016/j.cjcpc.2025.02.003

**Published:** 2025-03-08

**Authors:** Kota Nagaoka, Taisuke Nabeshima, Hitoshi Mori, Takuro Kojima, Ritsushi Kato, Naokata Sumitomo

**Affiliations:** a Department of Pediatric Cardiology, Saitama Medical University International Medical Center, Hidaka, Saitama, Japan; b Department of Cardiology, Saitama Medical University International Medical Center, Hidaka, Saitama, Japan

**Improved prognosis after congenital heart disease surgery has led to an increase in aging patients at risk of atrial arrhythmias.****^1^** **Accessing the pulmonary venous atrium (PVA) after Fontan surgery is challenging. A puncture at the transcaval cardiac area between the inferior vena cava and PVA has been performed in a small number of cases.****^2^** **However, the narrowness of this region made it challenging to perform a safe puncture in this area.****^3^** **In this paper, we report a case in which we successfully and safely accessed the PVA by visualizing the narrow transcaval cardiac area using a SOUNDSTAR catheter.**

## Case Report

**Figure 1:**
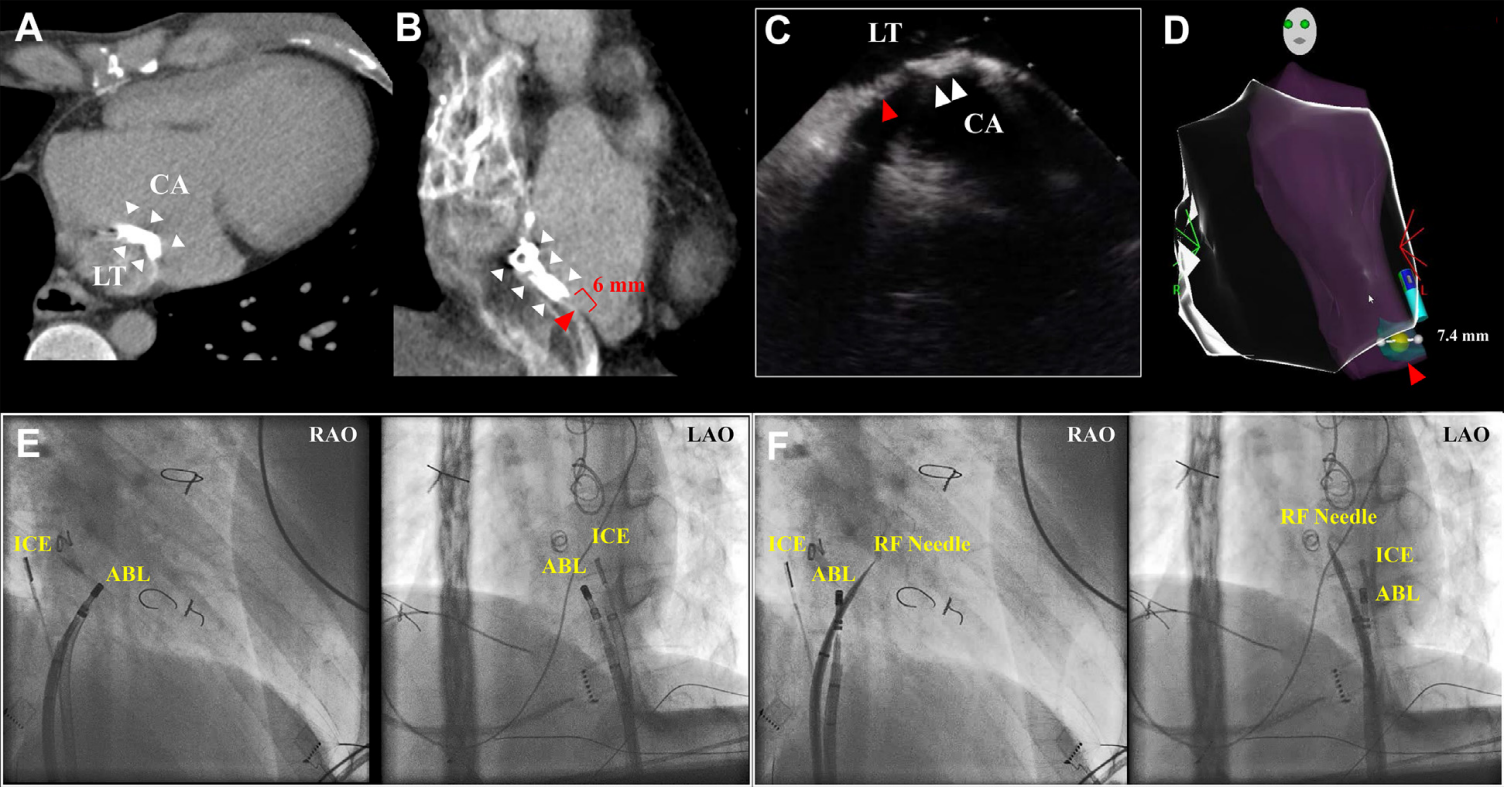
(**A**, **B**) Computed tomography before the ablation. A highly calcified lateral tunnel (LT) baffle (**white arrowhead**) and adjacent native tissue whose length was 6 mm (**red arrowhead**) were observed. (**C**, **D**) Intracardiac echocardiography and the geometry constructed with SOUNDSTAR. A small normal myocardium channel (**C**, **red arrowhead**) adjacent to the highly calcified baffle was observed. A 3D map of the LT (**D**, **pink area**) and normal myocardial area (**D**, **light green area**) was created using SOUNDSTAR. The width of the normal myocardial area was 7.4 mm. That point is marked on the 3D map. (**E**) Using an ablation catheter, the puncture site was marked on the fluoroscopic image based on the 3D mapping. (**F**) Based on the marked site of the ablation catheter, a transbaffle puncture was performed using a radiofrequency needle. ABL, ablation catheter; CA, calcification; ICE, intracardiac echocardiography; LAO, left anterior oblique: RAO, right anterior oblique; RF, radiofrequency transbaffle.

The case was a 28-year-old man with right isomerism, levocardia, left-sided inferior vena cava (IVC), single atrium, single ventricle, common atrioventricular valve, pulmonary artery stenosis, and total anomalous pulmonary venous return of the paracardiac type. He underwent a Fontan surgery using the lateral tunnel (LT) technique with expanded polytetrafluoroethylene (ePTFE) for the intra-atrial baffle during childhood. He had a history of multiple cardioversions for atrial flutter with a heart rate of 160 beats per minute under oral bepridil therapy, which led to his admission for catheter ablation of atrial flutter. Contrast computed tomography (CT) was performed before the ablation to investigate the previous operation, revealing a significant calcification in the LT, which posed a challenge for puncturing the intra-atrial baffle used to access the pulmonary venous atrium (PVA). On a detailed CT examination, a normal myocardial region was identified, which was approximately 6 mm in size, adjacent to the IVC at the lower end of the LT and in contact with the PVA (Fig. 1, A and B). Based on this finding, we performed catheter ablation using this narrow channel to access the PVA.

**Novel Teaching Points**

• Catheter ablation in adult patients after the Fonton operation can sometimes be challenging due to the anatomic limitations after its surgery.
• Puncture to the narrow cavoatrial area under 2-dimensional guidance using fluoroscopic imaging and intracardiac echocardiography is challenging.
• Combining intracardiac echocardiography with a 3-dimensional mapping system is useful for visualizing the exact location of the narrow channel in 3 dimensions.

We performed catheter ablation under the general anesthesia using a three-dimensional (3D) mapping system (CARTO 3; Biosense Webster, Irvine, CA). An 8 F long sheath, an 8.5 F braded sheath (SL0; Abbott, St. Paul, MN), and an 8.5 F steerable sheath (Agilis; Abbot) were secured from the right femoral vein. Using the SOUNDSTAR catheter (SOUNDSTAR; Biosense Webster), we identified noncalcified normal tissue adjacent to the PVA on the IVC side of the calcified LT tunnel using intracardiac echocardiography images (Fig. 1C), as with the preoperative CT scan. The LT geometry was first created (Fig. 1D). We delineated the normal tissue area using SOUNDSTAR and marked the puncture site with a yellow tag (Fig. 1D, yellow tag). Using 3D mapping guidance, we inserted the ablation catheter through the Agilis sheath and positioned it at the site marked with the yellow tag. Fluoroscopic images of this ablation catheter were recorded at that site (Fig. 1E). Subsequently, the SL-0 sheath was positioned at the marked point under fluoroscopic guidance. Without this marking, accurately placing the SL-0 sheath in the transcaval area, which is very small, would have been challenging using only intracardiac echocardiography guidance. After placing the SL-0 sheath at the puncture site (Fig. 1F), we used intracardiac echocardiography imaging to ensure its precise visualization at the intended location and performed the transcaval cardiac puncture using a radiofrequency transbaffle needle (RF needle; Boston Scientific, Marlborough, MA) that the puncture needle had safely reached the PVA, we subsequently passed the ablation catheter through the same puncture site. The SL-0 sheath and Agilis sheath were then positioned in the PVA.

**Figure 2:**
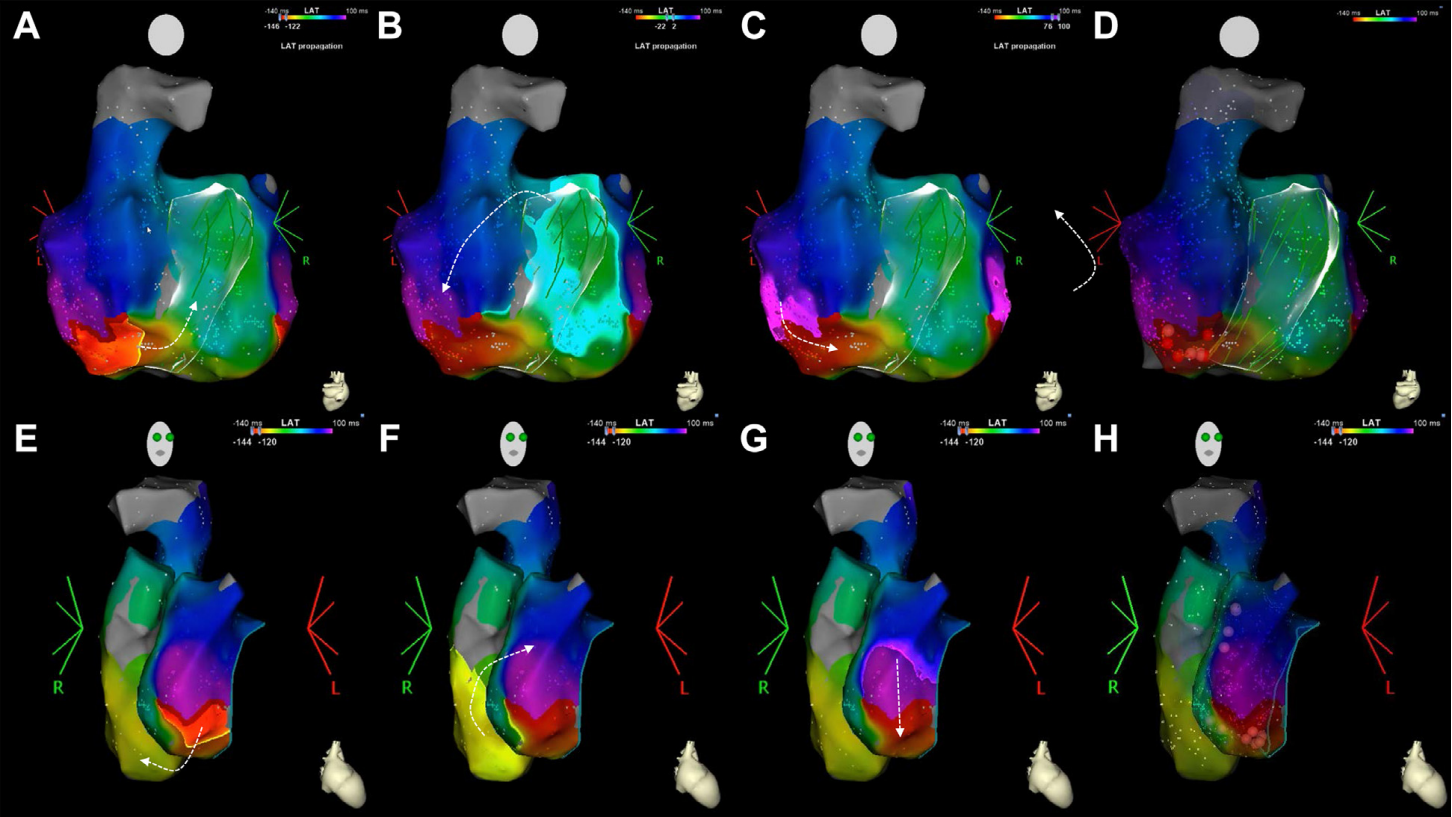
Activation mapping of the pulmonary venous atrium (PVA) and LT. AT1 showed a counterclockwise rotation pattern around the PVA (**A**-**D**), and AT2 showed a clockwise rotation pattern around the PVA that included the LT as part of the circuit (**E**-**H**). The **red tag** indicates the ablation site. Ablation was performed on the inferior part of the AT1 circuit, followed by the anteroinferior LT site, which resulted in the termination of the tachycardia. AT, atrial tachycardia; LAT, local activation time; LT, lateral tunnel.

We positioned a 2 F 8-pole electrode catheter (EPstar; Japan Lifeline, Tokyo, Japan) in the left pulmonary artery as a reference catheter from the right internal jugular vein and a 4 F 4-pole electrode catheter (Inquiry; Abbott) in the single ventricle via the aortic valve. On atrial extrastimulation, 2 types of tachycardia were induced: one with a cycle length (CL) of 286-300 ms (AT1, Video 1 , view video online) and another with a CL of 324-347 ms (AT2, Video 2 , view video online). These tachycardias readily alternated. Both tachycardias exhibited atrioventricular dissociation on administration of adenosine triphosphate, leading to a diagnosis of atrial tachycardia (AT1 and AT2, respectively). Using a 5-spline high-resolution mapping catheter (PENTARAY; Biosense Webster), an activation map of the PVA was created. In the activation map, a counterclockwise rotation pattern around the PVA was observed (Fig. 2, A-D). With the addition of LT mapping, a reentry pattern that included the LT as part of the circuit was also identified, indicating that this tachycardia was a dual loop tachycardia (Fig. 2, E-H). Entrainment pacing from the anteroinferior direction of the LT exhibited a postpacing interval of 325 ms, which matched the tachycardia CL of AT2 and indicated that the circuit of AT2 included the LT myocardium. On the basis of the activation map, we first ablated the inferior part of the AT1 circuit in the PVA (Fig. 2D), resulting in the tachycardia changing to the AT2 only. Then we ablated the anteroinferior LT site of the AT2 circuit, resulting in the termination of the tachycardia. The procedure was concluded because we confirmed that no further tachycardia was induced via atrial or ventricular pacing.

## Discussion

In catheter ablation of supraventricular tachycardia after a Fontan surgery, approaching the PVA is required in many cases. For puncture of the extracardiac conduits, a metal needle is typically used, and sometimes a specific technique involving a snare may be required.^4^ A metal needle is also used in cases involving an LT with an ePTFE baffle, whereas an RF needle is selected if the intra-atrial baffle is created using self-pericardium tissue without strong calcification.^4^ However, the incidence of calcification in ePTFE material used for extracardiac conduits in total cavopulmonary connection has been reported to be high.^5^ In such cases, transcaval cardiac puncture, an approach involving the puncture of the overlapping area between the IVC and the PVA, has been reported to be effective.^2^ However, the median length of this overlapping area has been reported to be 11.7 mm (6.8-14.0 mm),^3^ making the puncture more challenging. In our case, being an LT with a calcified ePTFE patch rather than an extracardiac conduit type Fontan, the transcaval cardiac area by CT was approximately 6-7 mm, narrower than previously reported. Previous reports have described performing punctures under fluoroscopic and echocardiographic guidance; however, these images were 2D, and 3D analysis was necessary to accurately identify the narrower channel.^6^ We previously reported a case with high calcification of the interatrial septum after an atrial septal defect operation.^7^ In this report, the septal puncture was safely performed by identifying the narrow puncture site using the SOUNDSTAR and 3D mapping system. In the present case as well, a combination technique using intracardiac echocardiography and a 3D mapping system successfully visualized the narrow channel, allowing the puncture to the PVA to be safely performed without any complications.

After the Fontan surgery in patients, as the patient grows, the length of the transcaval cardiac area can expand, creating areas where the IVC comes in contact with the atrial muscles. This phenomenon is recognized as one of the options for the puncture site selection.^3^ In cases where the length is sufficient, safe puncture can be performed under fluoroscopic guidance. However, in cases like this one, where the length is shorter, the puncture becomes more challenging. Our marking technique with SOUNDSTAR is useful to identify the puncturable site in the 3D mapping, ensuring a safe puncture without complications.

## Conclusions

We present a successful case of catheter ablation after an LT Fontan operation with a narrow length of the transcaval cardiac area with the calcified LT. Our puncture method, where the puncture site is marked on the 3D mapping, would be a useful technique for patients with a narrow channel after a congenital heart disease operation, particularly those with a complex cardiac structure.

## Supplementary Material

Video 1 Activation mapping of the AT1. AT1 showed a counterclockwise rotation pattern around the pulmonary venous atrium. All ablation sites are visualized as red tabs. AT, atrial tachycardia.

Video 2 Activation mapping of the AT2. AT2 showed a clockwise rotation pattern around the pulmonary venous atrium that included the lateral tunnel as part of the circuit. AT, atrial tachycardia.
